# Preterm Birth: An Overview

**DOI:** 10.7759/cureus.33006

**Published:** 2022-12-27

**Authors:** Vivekanand Khandre, Jyotsana Potdar, Akshunna Keerti

**Affiliations:** 1 Obstetrics and Gynaecology, Jawaharlal Nehru Medical College, Datta Meghe Institute of Medical Sciences, Wardha, IND; 2 Medicine and Surgery, Jawaharlal Nehru Medical College, Datta Meghe Institute of Medical Sciences, Wardha, IND

**Keywords:** transvaginal ultrasound, short cervical length, progesterone, prematurity, preterm birth

## Abstract

Birth order has a significant impact on perinatal and long-term outcomes. Preterm birth rates, ranging from 5% to 18%, are regrettably still high in industrialized and developing countries, making them the main contributor to infant mortality and morbidity. Infection, cervical pathology, uterine overdistension, progesterone deficiency, stress on the mother and fetus, allograft reaction, allergic phenomena, and likely more unknown factors are just a few of the causes of preterm birth syndrome. These several causes may improperly stimulate the usual pathway between the decidua and the fetal membranes, resulting in cervical ripening, membrane rupture, and uterine contractility. Some of the mechanisms underpinning these actions include receptors, chemokines, and inflammatory cytokines. For early identification, treatment, and avoidance of negative consequences, it is essential to understand the cellular and metabolic mechanisms that cause preterm labor. Clinicians and researchers are crucial to improving our knowledge of the biochemistry of preterm delivery, identifying risk factors, and creating treatments for this challenging condition. Intrauterine growth restriction and pre-eclampsia or eclampsia are frequent causes of suspected preterm births. “Spontaneous preterm births” occur after preterm labor that develops without warning with an early membrane rupture. It is thought that the condition that may cause these births may have several causes, such as uterine overdistension, vascular disease, infection, or inflammation. Unplanned preterm births have several reasons, including the black race, periodontal disease, low mother body mass index (BMI), and previous preterm births. A short cervical length and a high cervical-vaginal fetal fibronectin concentration are the two best signs of premature birth.

## Introduction and background

Premature births occur before the 37th week of pregnancy, though local variations exist in the low-gestational age cutoff that distinguishes them from spontaneous abortion [1]. Preterm deliveries cause more than half of long-term morbidity and 75% of perinatal mortality. Preterm newborns are more prone to suffer from respiratory, gastrointestinal, and cognitive issues even though they often survive. Rising indicated preterm births are primarily responsible for the singleton preterm birth rate [2]. The rise in preterm births is mainly caused by multiple preterm gestations brought on by assisted reproductive technologies. Premature birth is more likely to occur in singleton pregnancies after in vitro fertilization [3]. Preterm premature rupture of the membranes, or PPROM, is a spontaneous rupture that happens less than 37 weeks into the pregnancy and at least one hour before labor begins. Even though the origin is usually unclear, asymptomatic intrauterine infection is typically preceded by membrane rupture [4]. Although infections and smoking have a significant impact, the risk factors for PPROM are essentially the same as those for premature spontaneous labor with intact membranes. Within a few days, most PPROM patients spontaneously go into labor. However, many patients must wait weeks or even months before giving birth [5]. Because the membranes typically serve as a barrier to ascending infection, intrauterine infection and premature labor are frequent complications of PPROM [6]. Regular contractions and cervical change occur at a gestational age of fewer than 37 weeks, referred to as preterm labor [7]. The cause of premature labor is unknown; it might be brought on by pathogenic trauma or the idiopathic beginning of the normal labor process. The idea that the fetus plays a part in determining when labor starts have been supported by research on sheep [8]. Parturition in sheep cannot start until fetal cortisol is present because it is possible to remove the fetal hypophysis, the adrenal glands, or both [9]. Fetal cortisol is necessary for labor to begin.

Progesterone withdrawal, oxytocin initiation, and decidual activation are three theories for the start of term labor [10]. Progesterone has been eliminated due to research on sheep [11]. The fetal-adrenal axis responds positively to the adrenocorticotropic hormone as parturition approaches, increasing cortisol production [12]. The activity of the placental 17-hydroxylase is stimulated by fetal cortisol, which reduces progesterone release and boosts estrogen production. Prostaglandin synthesis is increased when the estrogen/progesterone ratio is changed, which starts a chain of events that leads to labor [13]. As labor proceeds, serum progesterone levels in humans do not fall. However, since progesterone antagonists, like RU486, start preterm labor, and progestational drugs stop it, lowering local progesterone levels or the number of receptors is a likely mechanism for labor to begin [14]. Because oxytocin increases uterine contraction frequency and force, it is thought to have a role at the beginning of labor. Because blood oxytocin levels do not rise before labor and oxytocin clearance does not vary, oxytocin is unlikely to initiate labor [15].

## Review

Methodology

The databases PubMed, Medline, Scopus, Embase, and Web of Science were used in the literature search. Transvaginal ultrasound, short cervical length (CL), progesterone, premature birth, and other search phrases were employed. They were included if studies matched our predetermined inclusion criteria, such as premature birth. Our review did not include abstracts, conference articles, systematic reviews, or meta-analyses. There were no restrictions on the publishing date of articles, and only those written in English were considered.

Causes

Infection or inflammation, ischemia or bleeding in the uteroplacental, uterine overdistension, stress, and other immunologically generated processes are a few causes of preterm labor [16]. Since a precise mechanism is usually impossible to identify, explanations for premature labor have instead searched for factors connected to preterm delivery but not necessarily in the causation pathway [17]. Preterm labor, or PPROM, is hypothesized to develop due to an interaction between increasing risk factors [18]. Higher activation of the infection or inflammatory pathway may assist in explaining some of the increases in preterm deliveries linked to different risk factors, as multiple risk factors enhance systemic inflammation [19]. Table 1 below shows categories of preterm birth [20].

**Table 1:** Categories of preterm birth [20]

Category	Gestational (weeks)
Extremely preterm	<28
Very preterm	28-32
Moderate preterm	32-37
Early moderate preterm	32-34
Late moderate preterm	34-37

Congenital deficiencies are frequently caused by exposure to certain medicines, sometimes known as teratogenic drugs, and are one of the major causes of newborn death. Congenital disabilities-also is known as teratogenic conditions-have a high probability of developing due to exposure to certain chemicals, which is one of the main factors in infant mortality [21]. Preterm birth is more likely in pregnant women with a history of drug abuse or dependence. This risk applies to all the drug categories that were looked at, including opioid, cocaine, cannabis, amphetamine, and other or polysubstance types, with cocaine users showing the highest risk-nearly one in four had premature births. All classes, except cannabis, had a higher chance of early term delivery related to drug addiction or dependency [22]. Of all the subtypes of preterm delivery, cocaine and polysubstance use in women was associated with the most significant risk. Chronic hypertension and diabetes are becoming more common, including an increase in the prevalence of diabetes-complicated hypertension. It is also well-established how each of these diseases affects prenatal adverse outcomes.

It makes sense to define risk factors for predicting preterm delivery for several reasons. Finding at-risk women is the initial stage, after which risk-specific therapy may start. Second, the risk factors could assist in pinpointing a population for study on specific treatments. Finally, determining risk factors may offer vital insight into preterm birth mechanisms. Preterm birth has been associated with a wide range of maternal or fetal characteristics, including the mother's demographics, nutritional status, pregnancy history, current pregnancy characteristics, psychological traits, negative behaviors, infection, uterine contractions, CL, and biochemical and genetic markers [23].

Premature birth is more likely to occur in pregnancies that are near in time to an earlier delivery. After adjusting for confounding factors, an interpregnancy gap of fewer than six months is more significant than a risk of premature delivery that is two times greater [24]. A small opening is also considerably more common in women with preterm first deliveries than those with term first births, increasing the risk. Even though the exact mechanism is unknown, the uterus needs time to get back to normal, especially to stop being inflamed from the previous pregnancy [25]. Maternal depletion may also be at work because pregnancy depletes the mother's essential vitamins, minerals, and amino acids [26]. The likelihood of resupplying these nutrients is lower after a brief pause.

Biological and genetic markers

To assess how well biomarkers predict preterm birth, saliva and other biological fluids (such as amniotic fluid, urine, cervical mucus, vaginal secretions, serum or plasma, or both) have been used. Numerous analytes, particularly those connected to inflammation, have been measured, including cytokines, chemokines, estrogen, and others, and many of them have been linked to premature birth [27]. Studies of biomarkers have advanced our knowledge of the underlying mechanisms causing spontaneous preterm birth, even though few biomarkers have shown evidence of therapeutic benefit. Determining the analyte’s collection and transport period is just as important as knowing its specifics and where it originated from. For instance, around 24 hours before labor begins, the serum content of matrix metalloproteinase-9 considerably increases. Despite being of limited use in the prevention, such late prediction can help understand the pathophysiology of preterm labor. Although salivary oestriol concentration is not very practical for predicting early preterm deliveries, it does predict late preterm birth rather well. The minimal morbidity associated with late preterm infants indicates that these deliveries are of limited significance [28].

Biochemical markers found in bodily fluids, such as amniotic fluid, urine, cervical mucus, vaginal secretions, serum or plasma, and saliva, have been used to detect preterm labor. A fetal fibronectin glycoprotein may be discovered in the decidua basalis’ extracellular fluid near the intervillous space [29]. Between 18 and 34 weeks of gestation, it is often present in the vagina in low quantities, and its presence has served as a helpful indicator of a pathologic disturbance of the maternal-fetal interface. Positive values range from 50 ng/mL and above. C-reactive protein (CRP), a marker of maternal systemic inflammation, has been investigated as a preterm delivery prediction. Because of the inflammatory response from the germs that travel from the vagina to the uterus during pregnancy, interleukin-6 production rises [30]. Preterm labor starts when the decidua, chorion, and amniotic fluid produce prostaglandins and matrix metalloproteinases (MMPs). Cervical IL-6 levels were more significant in women who delivered at fewer than 32 weeks gestation than in those who did not. Alpha-fetoprotein (AFP) elevation at 24 weeks is linked to a higher risk of premature labor starting. Estriol is the primary type of estrogen that circulates in the body during pregnancy, and measurements of this molecule from samples of the mother’s saliva seem to match her serum levels [31]. Clinically, early estriol rise or elevated levels (2.3 ng/mL) may help identify women more prone to premature labor and delivery. Premature labor is more likely when there is high beta-human chorionic gonadotropin (beta-hCG) in cervicovaginal fluid, typically appearing in the early second trimester.

Diagnosis

Numerous studies have looked at placental alpha microglobulin-1 (PAMG-1) to determine if it may predict future spontaneous preterm birth in women who had early labor signs, symptoms, or complaints [32]. The PAMG-1 test, also known as the PartoSure test, is the most important predictor of impending spontaneous birth within seven days of a patient presenting with preterm labor signs, symptoms, or complaints, according to one study. It is in contrast to fetal fibronectin tests and transvaginal ultrasound assessments of CL [33]. The assays’ relative positive predictive values (PPVs) for PAMG-1, fetal fibronectin (fFN), and CL were 76%, 29%, and 30% (P < 0.01) [34-37]. fFN has emerged as a critical biomarker; its presence in cervical or vaginal secretions signals a breakdown of the chorion-decidua barrier [38]. A negative test has a high probability of being accurate, but a positive test raises the chance of an early birth [39]. Only 1% of mothers who were in suspected preterm labor and the test came back negative gave birth to their infants the next week, it was found [40]. Women whose cervixes potentially result in an early delivery have had their cervixes evaluated by obstetric ultrasonography. The most typical indicator of cervical incompetence is a CL of less than 25 mm at or before 24 weeks of gestation, making it undesirable to deliver a baby preterm with a small cervix [41].

Prevention and treatment

Experience with assisted reproduction shows that when the number of embryos transplanted is reduced, particular professional techniques can quickly reduce the risk of preterm birth. Folic acid ingestion before pregnancy is indicated to avoid congenital disabilities. Additionally, research suggests that folic acid supplements taken before conception (i.e., before becoming pregnant) may lessen the likelihood of preterm births. For expecting moms and their offspring, quitting smoking is beneficial [42]. Controlling preterm birth risk factors, obtaining the appropriate medical care, managing preterm birth risk factors, and eating healthfully are examples of self-care practices that reduce the risk of premature delivery (such as working long hours on your feet, being exposed to carbon monoxide, experiencing domestic abuse, and other factors). The chance of a preterm delivery does not appear lessened by reducing physical activity during pregnancy. Healthy eating habits, such as dietary adjustments and taking prescription vitamins, can be advantageous at any stage of pregnancy. Only a small number of adverse outcomes, such as preterm birth, pre-eclampsia, and maternal death, may be avoided by supplementation in women with insufficient dietary calcium [43].

Pyelonephritis and the chance of having a baby too soon are decreased by checking for asymptomatic bacteriuria and getting the proper treatment. According to preliminary data, determining the cervix length in preterm labor patients can aid treatment modifications and lead to a pregnancy extension of roughly four days [44]. Regular ultrasound exams of the cervix can detect pregnant women at risk for premature labor. It is not advisable to test for fibronectin in vaginal secretions in women with a low risk of delivering too soon. It is possible to tell if a woman is more likely to give birth early by looking at her prior obstetrical history or seeing if she possesses any known risk factors. For certain people, preconception intervention provides several advantages. Optimizing medication therapy before conception may be advantageous for patients with certain medical conditions, including diabetes, hypertension, asthma, and others [45].

Surgery may be required to treat some uterine anomalies in patients (i.e., excision of a uterine septum). Premature birth is far more likely to occur in multiple pregnancies, typically resulting from assisted reproductive technologies [46]. To decrease the number of fetuses to two or three, selective reduction is performed. Progestogens have anti-inflammatory qualities, relax the uterine muscle, and are frequently provided as vaginal progesterone or hydroxyprogesterone caproate. These effects are thought to cause physiological and anatomical changes that are advantageous in lowering preterm birth. While bacterial vaginosis in pregnancy may be treated with antibiotics, this does not seem to alter the risk of premature delivery. Antibiotics may not be able to alleviate the requirement for premature birth in chronic chorioamnionitis since they are insufficient for treating the illness [47]. The woman’s cervix shortens as she gets closer to giving birth. Ultrasonography can identify premature cervical shortening, which is associated with preterm delivery. During cervical cerclage surgery, a suture is wrapped around the cervix to stop the cervix from shortening and expanding.

Tertiary interventions focus on women who are about to enter preterm labor, rupture their membranes or bleed early. Combining ultrasonography with the fibronectin test increases diagnostic precision and decreases false-positive findings. While early labor interventions may not be able to give the fetus enough time to continue growing and maturing in cases of progressive cervical dilatation and effacement, they may be able to postpone delivery long enough for the mother to be transferred to a facility that is prepared with the necessary tools and is trained to handle preterm births [48]. To keep patients hydrated, hospitals use intravenous infusions (as dehydration can lead to premature uterine contractions). If a newborn has cardiac arrest during birth and is under 400 g, is delivered before 23 weeks, or both, resuscitation attempts are not advised. The likelihood that the baby would catch group B streptococcus and the incidence of deaths associated with the condition are both shown to be decreased when all pregnant women at risk of threatening preterm labor get regular antibiotic medication [49]. Extremely preterm infants may have undeveloped lungs because their surfactant is not yet produced. Hyaline membrane disease sometimes referred to as respiratory distress syndrome in newborns can result from this. Glucocorticoids, a prenatal steroid that penetrates the placental barrier and stimulates the synthesis of surfactant in the baby’s lungs, are commonly administered to pregnant women who face a possible preterm delivery before 34 weeks to reduce the probability of this event. The American Congress of Obstetricians and Gynecologists advises against using steroids after 37 weeks [50]. The typical glucocorticoids used in this situation are betamethasone or dexamethasone, frequently administered after the pregnancy reaches viability at 23 weeks.

## Conclusions

Since preterm delivery is one of the leading causes of death and neurodevelopmental problems in children under five worldwide, it continues to be a critical public health concern. The most common cause of mortality for children under the age of five globally is preterm delivery, but with developments in technology and medicine, more babies born just before they are no longer viable are surviving. With increased survival, long-term neurologic impairments are more likely to develop. Preterm delivery, a severe healthcare concern, affects 15 million infants annually. The March of Dimes and other advocacy organizations’ research and lobbying activities have brought preterm birth prevention to the public’s notice. However, there is compelling evidence that preterm birth rates are rising globally and in most countries.
